# Function and evolution of RNA N6-methyladenosine modification

**DOI:** 10.7150/ijbs.45231

**Published:** 2020-04-15

**Authors:** Zhi-Man Zhu, Fu-Chun Huo, Dong-Sheng Pei

**Affiliations:** Department of Pathology, Xuzhou Medical University, Xuzhou 221004, China

**Keywords:** m^6^A modification, methylation, RNA, function

## Abstract

N6-methyladenosine (m^6^A) is identified as the most prevalent and abundant internal RNA modification, especially within eukaryotic mRNAs, which has attracted much attention in recent years since its importance for regulating gene expression and deciding cell fate. m^6^A modification is installed by RNA methyltransferases METTL3, METTL14 and WTAP (Writers), removed by the demethylases FTO and ALKBH5 (Erasers) and recognized by m^6^A binding proteins, such as YT521-B homology YTH domain-containing proteins (Readers). Accumulating evidence shows that m^6^A RNA methylation participates in almost all aspects of RNA processing, implying an association with important bioprocesses. In this review, we mainly summarize and discuss the functional relevance and importance of m^6^A modification in cellular processes.

## Introduction

To date >170 chemical modifications in RNA have been found in organisms 1. N6-methyladenosine (m^6^A) modification was firstly discovered in the early 1970s from cancer cells. This modification also exists in various species, including yeast, Arabidopsis, Drosophila, various viruses, zebrafish, plants, fruit flies, mice, and humans 2. In 2012, Dominissini et al. revealed the m^6^A distribution in human and mouse and identified more than 12000 methylated sites on human mRNAs by utilizing m^6^A-seq 3. m^6^A sites are highly conserved and generally enriched in the consensus motif RRACH (R = G or A and H = A, C, or U), which is more prone to be detected in the 3'-untranslated regions (3'UTRs), near stop codons and within internal long exons 3.

m^6^A methylation is introduced into RNAs by a multicomponent methyltransferase complex (m^6^A writers). The complex traditionally consists of methyltransferase-like 3 (METTL3), METTL14 and Wilms'tumor 1-associating protein (WTAP), which effectuates the m^6^A methylated group into RNAs. Subsequently, new writers, such as RBM15(B), HAKAI, METTL16, KIAA1429 (VIRMA) and ZC3H13 have been identified 1, 4. METTL3 serving as the core component and METTL14 recognizing target RNAs integrate a stable heterodimer complexes referring to interacting with other m^6^A cofactors to synergistically catalyze m^6^A methylation 5. WTAP contributes to the methyltransferase complexes anchoring in nuclear speckles. m^6^A methylation can be removed by RNA demethylases (m^6^A erasers), fat mass and obesity-associated protein (FTO) and AlkB family member 5 (ALKBH5). FTO, the first identified m^6^A demethylase, oxidizes m^6^A in RNA to N6-hydroxymethyladeosine and N6-formyladenosine 6. ALKBH5, an FTO homologue, directly abrogates m^6^A modification to adenosine without intermediate detected 7. Although the m^6^A modification is dynamically regulated by writers and erasers, the proteins (m^6^A readers) preferentially recognizes m^6^A-modified sites, influencing RNA fate and endowing distinct biological functions. m^6^A readers, mainly including YT521-B homology (YTH) domain family proteins (YTHDF1~3), YTH domain containing proteins (YTHDC1~2), IGF2BP1~3, HNRNPC/G/ A2B1 and eIF3, regulate RNA processing, structure, nuclear export, translation and degradation. YTH domain, as m^6^A binding module, shares a conserved α/β fold and can discriminate between non-modified and m^6^A mRNAs 8, 9. YTHDF1 can bind to the 3'UTRs and stop codon of m^6^A-containing RNA and promotes translation initiation by interacting with eIF3 10. Binding sites of YTHDF3 also primarily locates in 3'UTR 11. YTHDF2 associates with half-life of mRNA. YTHDC1 regulates transcription of target genes and alternative splicing of mRNA 12. HNRNPC regulates mRNA structure, while HNRNPA2/B1 involves pre-miRNA transcription 13. IGF2BPs enhances mRNA stability and storage. Reader proteins combine m^6^A methylation with RNA processing and biological functions.

m^6^A modification characterized by wide existence, unique distribution and dynamic reversibility. m^6^A methylation regulatory network regulates RNA processing and metabolism and participate in many cellular biological processes, such as immune modulation, fat metabolism, biological rhythm, reproductive development, and its disorders can cause various diseases. In this review, we summarized the current knowledge on the function and biology of m^6^A methylation.

## Detection of m^6^A methylation

Due to technical bottlenecks, it makes the m^6^A methylation more mysterious and incomprehensible. m^6^A modification neither modulates reverse transcription nor is analogous to m^7^G methylation characterized by being specifically cleaved, the presence of m^6^A distributing and differential patterns at a particular mRNA is challenging to observe and detect 14, 15. m^6^A methylation has not been characterized until the availability of transcriptome-wide mapping approaches, m^6^A-seq and MeRIP-seq 3, 15, 16, both of which capture m^6^A RNA fragments through immunoprecipitation and then identify modified sequences.

Based on the theory, researchers detect an enormous amount of highly conserved m^6^A sites and also determine over 12,000 m^6^A signal peaks on 7,676 mammalian genes 3, 17. Nevertheless, the above detection analysis is insufficient to discriminate two adjacent m^6^A sites, and m^6^A mapping methods localize m^6^A residues to about 100~200 nucleotides, which may not accurately identify m^6^A sites in a whole transcript 18. Besides, both m^6^A-seq and MeRIP-seq may misread m^6^A_m_ modification that occurs at the 3'UTR ends of mRNA and is analogous to m^6^A modification containing the sixth methyl group as m^6^A methylation. MeRIP-Seq can identify m^6^A-modified sites in mammalian cells, whereas the way which is complex and only separate the m^6^A-abundant regions. Considering the above defects, researchers have made improvements in detection techniques for m^6^A methylation site. Ultraviolet cross-linking immunoprecipitation technology, including miCLIP 19, PA-m^6^A-seq 20, m^6^A-CLIP (also called UV-CLIP) 14, 21, are reported to overcome the defects above, which could discriminate m^6^A methylation at an individual- nucleotide resolution more accurately and provide higher resolution transcriptome-wide maps of m^6^A methylation. Another technique, m^6^A-LAIC-seq, introduces spike-in RNA as an internal reference founded upon m^6^A-seq and then calculates the methylation level of m^6^A modification at each gene fragment in a full transcriptome, however, it is incapable of determining single-base resolution of m^6^A modification 22.

In addition to high-throughput sequencing, monitoring of the m^6^A modification at an exact position of mRNA is also very essential. SCARLET, as the most common strategy, accurately maps a single m^6^A site in both lncRNAs and mRNAs and then determine the m^6^A methylation content in total RNA 23, 24. Albeit SCARLET with low-throughput and fancy features, the feasibility and accuracy allow SCARLET to be a frequently-used strategy for examining the accuracy of high-throughput detection of m^6^A modification. And SCARLET can also be allowed to screen other types of epigenetic modifications in RNA, such as m^5^C and Ψ modifications 25. Golovina et al. reported a strategy based on high-resolution melting analysis for monitoring m^6^A sites in a specific RNA position 26. Mechanistically, m^6^A methylation alters the melting temperature, which is indirectly measured by qPCR 26; however, whether it can be popularized remains to be verified. The recently proposed m^6^A-REF-seq provides a novel perspective for the identification of m^6^A single site, which eliminates the requirement for m^6^A specific antibody and makes it more suitable for the detection of minute and precious samples 27. In recent years, new auxiliary detection technologies continue to emerge, which further improve the accuracy, sensitivity and application range of the traditional high-throughput sequencing. The improvement of detection strategies contributes to profoundly comprehend the m^6^A modification and its associations with various pathophysiologies.

## Prediction of m^6^A methylation (site)

Since the identification of m^6^A methylation requires plenty of workforce and material resources, and it is rather dynamic and tissue-specific, bioinformatics prediction greatly improving research efficiency for the distribution and pattern of m^6^A methylation is developed. Zhang et al. firstly proposes the HIDDEN MARKOV MODEL to predict residual sites around known sites 28. Subsequently, pRNAm-PC and iRNA-Methyl (http://lin.uestc.edu.cn/server/iRNA-Methyl) for predicting m^6^A sites are developed, and the common characteristics of both are the application of support vector machine model 29, 30. According to this philosophy, Jia et al. develop RNA-methylPred method, a highly accurate predictor for identifying m^6^A sites, which is more efficient than before 31. Li et al. propose a better TargetM6A method, which can only predict the m^6^A sites in pri-miRNAs(http://csbio.njust.edu.cn/bioinf/TargetM6A) 32. Zhou et al. synthesize a variety of mathematical models and reports SRAMP predictor that is more effectively to predict m^6^A sites in mammalian RNAs 29. In 2018, a database website named RMBaseV2.0 was established, and it includes sequencing data of multiple epigenetic modifications of RNAs in 13 species (http://rna.sysu.edu.cn/rmbase/), including mass data on m^6^A methylation sites 33.

## Biological functions of m^6^A modification

**Figure 1:**
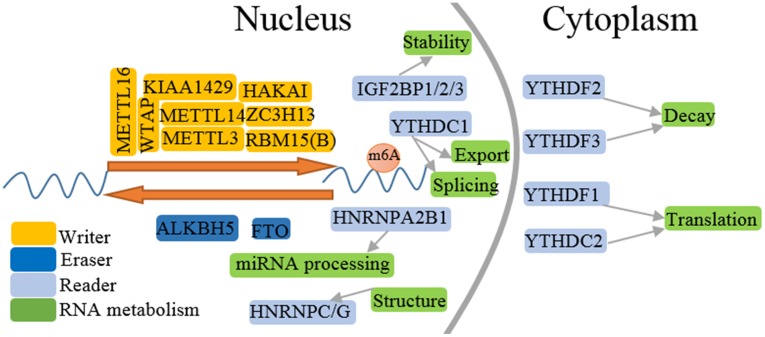
** Regtory process of m^6^A methylation.** m^6^A RNA methylation is installed by writers, reversed by erasers, and functionally facilitated by readers. m^6^A modification is involved in the life cycle of RNA metabolism including RNA splicing, structure, nuclear export, translation, degradation and miRNA processing.

**Figure 2:**
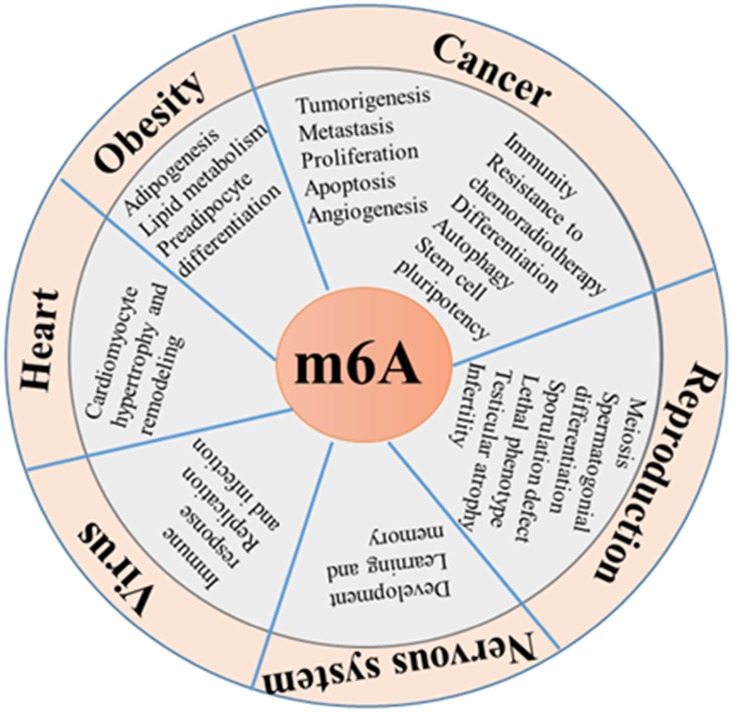
** Functional roles of m^6^A modification.** m^6^A modification plays important role in diverse biological processes, such as tumorigenesis, fat metabolism, biological rhythm, reproductive development, immune modulation, virus replication, cardiomyocyte remodeling and stress response.

**Figure 3:**
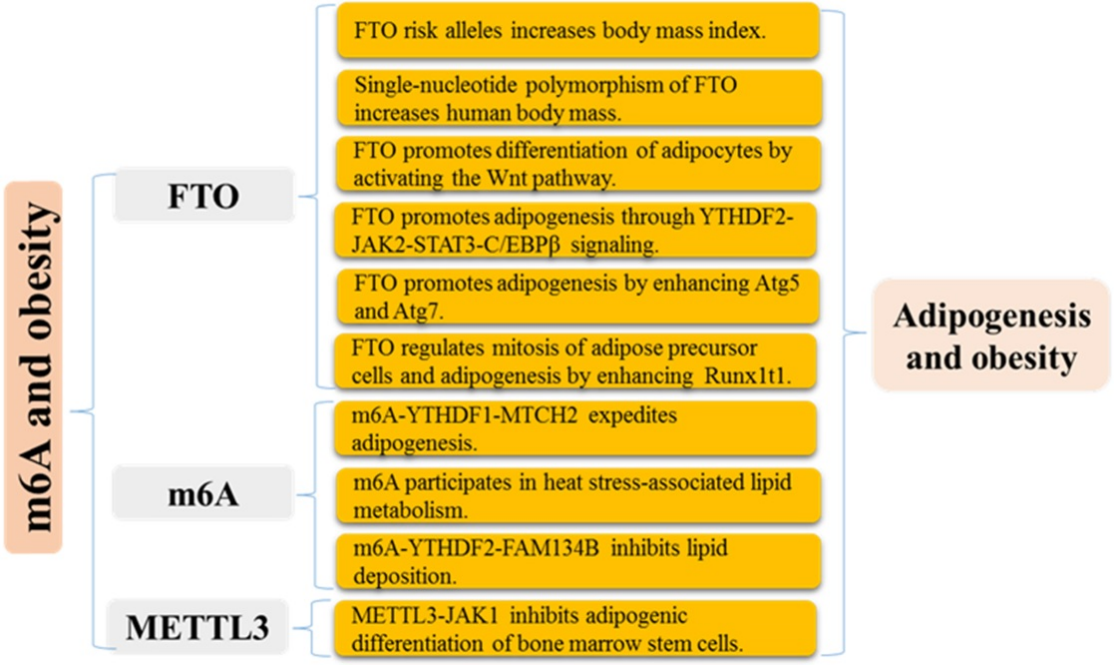
Mechanisms of m^6^A modification in adipogenesis and obesity.

**Figure 4:**
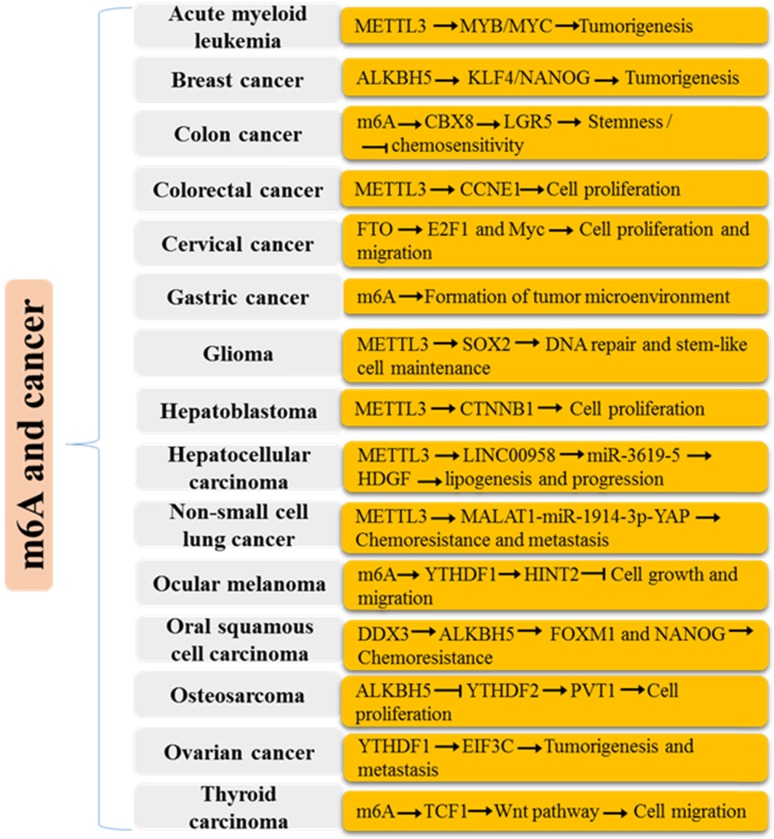
Mechanisms of m^6^A modification in various human cancers.

m^6^A functional enzymes cooperatively effectuates the dynamic balance of intracellular regulatory network m^6^A modification. Formidable evidence has revealed the important roles of m^6^A modification in various biological functions (Figure 1 and 2), such as RNA metabolism, adipogenesis (Figure 3), spermatogenesis, circadian rhythm, immune response, stem cell maintenance and tumorigenicity (Figure 4). Next, we mainly introduce several relatively well-studied roles of m^6^A modification in pathophysiological process.

### m^6^A in mRNA metabolism

The interaction between m^6^A modification and its regulators reconstructs dynamic regulatory networks, which is responsible for the mRNA processing and metabolism (Figure 1), from mRNA transcription, splicing and export in the nucleus to translation, localization and degradation in the cytoplasm, and further influencing gene expression, leading to various pathophysiologic processes.

### m^6^A and mRNA splicing

The process of pre-mRNA maturating into mRNA consists of 5' capping, 3' polyadenylation and splicing. Pre-mRNA splicing responsible for precise intron excision and exon connection is a necessary procedure for gene expression and enriches the diversity of gene products. m^6^A modification was initially considered as a splicing regulator, and prior research has revealed that it is more abundant in pre-mRNA than in mature mRNA 34. m^6^A modification is frequently enriched in the intron region of pre-mRNA 35, 36, its regulators primarily locate in nucleus speckle, a well-known location of pre-mRNA processing 37, and little methylation or demethylation occurs in cytoplasmic mRNA 38. Therefore, it can be inferred that m^6^A modification responsible for mRNA splicing occurs in the nucleus. Different effects of mRNA splicing accompany knockout of WTAP or METTL3, and WTAP is a known splicing factor 39. Inhibiting WTAP impairs mRNA levels and leads to abnormally alternative splicing of mRNA. METTL3 causes mis-splicing and instability of the mRNA 40. FTO silencing potentiates the splicing ability of SRSF2 and augments the number of target exons 41, 42. In addition, ALKBH5 has also been proved to affect splicing rates of mRNA 7. YTHDC1 can bind with SRSF3 and promote pre-mRNA splicing to mature mRNA 37. HNRNPC and HNRNPG are also responsible for pre-mRNA processing and mRNA maturation 43, 44. Remarkably, Ke et al. reported that m^6^A modification is not completely essential for the most mRNA splicing but acts as a determinant of cytoplasmic mRNA stability 38. Recently, it has been reported that m^6^A-assisted polyadenylation signals protect transcriptome integrity via exhibiting chimera formation in Arabidopsis thaliana RNA 45.

### m^6^A and mRNA localization

Nuclear export of mRNA is quite an important process to connect mRNA transcription and processing in nucleus with gene expression in cytoplasm. m^6^A modification contributes to nuclear export of mRNA, and attenuation of METTL3 reverses m^6^A modification and then jeopardizes the mRNA export 46. ALKBH5 depletion accelerates the mRNA translocation from the nucleus to the cytoplasm 7, whereas ectopic expression of ALKBH5 causes nuclear retention of mRNA of antiviral transcripts, thereby inhibiting transcription of mRNA transcripts 47. YTHDC1 recognizes m^6^A-modified mRNA and promotes its combining with nuclear RNA export factor NXF1 and transport adaptor SRSF3 to promote nuclear export 48.

### m^6^A and mRNA translation

METTL3 regulates mRNA translation through a different mechanism. By interacting with eIF3h, METTL3 promotes formation of densely packed polyribosomes and enhances translation of target mRNAs independently of its catalytic activity and m^6^A readers 48, 49. Stated differently, METTL3 localizing to the transcriptional start site frequently accompanied by CAATT-box binding protein CEBPZ of downstream genes induces m^6^A mRNA modification and augments translation via alleviating ribosome stalling 50. Through interacting ribosome and initiation factor, YTHDF1 binds to m^6^A -containing mRNAs and drives translation in an m^6^A-dependent manner 10, 51. On the other hand, YTHDF3 can also promote translation efficiency by combining with YTHDF1 and eIF4A3 52, indicating that YTHDF3 and YTHDF1 cooperate in the process of mRNA translation 53. Besides, IGF2BP1/2/3 can also accelerate mRNA translation 54.

### m^6^A and mRNA degradation

Reversing m^6^A with the blockade of METTL3 or METTLE14 alleviates degradation efficiency of mRNA and enhances gene expression 55. ALKBH5 regulates the stability of CYR61 mRNA and elevates its expression in trophoblast, which is deciphered by the mechanism that silenced ALKBH5 enhances the half-life of CYR61 mRNA 56. YTH domain in C-terminal of YTHDF2 binds to m^6^A-containing mRNA, while domains in N-terminal locate the mRNA to “RNA degrader” for further degradation 57. Recent studies reported that YTHDF2 destabilizes modified mRNA mainly by two distinct pathways, YTHDF2-CCR4/NOT complex and YTHDF2-HRSP12-RNase P/MRP complex 58. And YTHDF2-HRSP12-RNase P/MRP is also scalable for m^6^A-modified circRNA 58. HuR binds to the U-enriched region of 3'UTR of mRNA and jeopardizes the interaction the binding of miRNA to the Ago complex, preventing mRNA degradation 59. m^6^A modification impairs the binding ability of HuR and destabilizes m^6^A-containing transcripts. Similarly, blockade of METTL3 impedes the interaction between Ago2 and target mRNA, enhancing mRNA stability 10. What is of noteworthiness is that IGF2BP-mediated m^6^A “Reading” recently has been reported the promotive effect on mRNA stability, and IGF2BP may combine with mRNA-stabilizing proteins, such as HuR, MATR3 and PABPC1 to elevate the mRNA stability 52. In conclusion, m^6^A modification can regulate gene expression through modulating mRNA stability and degradation.

### m^6^A and mRNA structure

m^6^A modification can alter the mRNA structure and make it prone to combine with HNRNPC and HNRNPG. m^6^A methylation contributes unwind partial mRNA double-strand of MALAT1 and CDS2 to exposure single-stranded U sites, and HNRNPC can recognize specific sites to regulate gene expression 13. m^6^A methylation of MALAT1 hairpin strengthens accessibility of HNRNPG binding by its C-terminal low-complexity region rather than the conventional RNA binding domain 60. The effect of structural remodeling partly caused by m^6^A modification on the binding of mRNA to proteins is called “m^6^A switch”, which is ubiquitous in the transcriptome, and plays biological function by regulating the interaction between mRNAs and binding proteins 13.

### m^6^A in adipogenesis and obesity

Early studies revealed that ectopic expression of FTO enhances food intake and fat mass in mice 61, while blockade of FTO protects against fat accumulation 62, 63. FTO has been identified as a positive and essential regulator of preadipocyte differentiation 64-66. And accumulating evidence highlights the necessity for FTO/m^6^A/YTHDF2 axis in adipogenesis regulation 64, 65. FTO silencing accelerates the decay of JAK2 mRNA in an m^6^A-YTHDF2 dependent mechanism, which restricts STAT3 phosphorylation and C/EBPβ transcription and expression, suppressing adipogenesis, and treating with specific FTO inhibitor also eliminates the accumulation of lipid droplets 67. Loss of FTO impedes adipogenesis of 3T3-L1 preadipocyte via the prolongation of cell cycle progression in which YTHDF2 recognizes and destabilizes m^6^A-modified CCNA2 and CDK2 mRNAs, crucial cell cycle regulator 64. Consistent with this, epigallocatechin gallate, as upstream regulators, negatively regulates FTO/ m^6^A/YTHDF2 axis and induces adipogenesis inhibition in 3T3-L1 preadipocyte 65. The latest research indicated that METTL3 knockdown decreases m^6^A mRNA modification of JAK1, rather than JAK2, and impairs YTHDF2-mediated mRNA degradation, which activates JAK1/STAT5/C/EBPβ pathway and expedites adipogenesis of bone marrow stem cells in porcine 68. ZFP217 directly activates the FTO transcription by binding to its promoter and interacts with YTHDF2 to maintain FTO-induced m^6^A demethylation, thereby facilitating adipogenesis of 3T3-L1 cells 66. m^6^A methylation expedites adipogenesis of intramuscular preadipocyte through enhancing MTCH2 expression in a YTHDF1-dependent mRNA translation 69. Besides, m^6^A participates in heat stress-associated lipid metabolism, and its regulators including METTL14, WTAP, FTO, and YTHDF2 and m^6^A levels are increased in the liver and abdominal fat of neonatal piglets following heat stress exposure 70. In summary, m^6^A modification and its modulators, especially FTO, functionally regulate adipogenesis (Figure 3), which provides a new understanding for pathogenesis and treatment of obesity-associated diseases from the RNA epitranscriptome modification.

### m^6^A in embryonic development and sex determination

m^6^A modification can regulate the mRNA process in the initial stage of life, and its aberrant alterations will inevitably cause abnormal embryonic development. Abnormal m^6^A modification affects the formation of male and female gametes, or restricts the activation of zygote genes, leading to abnormal embryonic development, and influence the normal function of offspring.

METTL3 modulates the alternative splicing of spermatogenesis-related genes, which are responsible for male fertility and spermatogenesis, and deletion of which influences spermatogonial differentiation and hinders meiosis initiation in mice 40. In Saccharomyces cerevisiae, the knockdown of IME4, a homologous gene of METTL3, causes sporulation defects 71. In Arabidopsis thaliana, the inactivation of MTA, a homologous gene of METTL3, causes the formation of lethal phenotypes or the stagnation of seed development in the spherical phase 72. Analogously, in Drosophila melanogaster, IME4, is critical for the regulation of Notch signaling in follicular development 73. Furthermore, IME4 regulates meiotic DNA replication and promotes meiosis in yeast through downregulating RME1 (a transcriptional repressor of meiosis) mRNA levels in an m^6^A-dependent manner 74. Upon METTL3 knockdown in the immature embryonic stem cells of mice, the immature phenotype cannot be fully terminated because of the inability of m^6^A modification, leading to early embryonic death 75.

The expression of ALKBH5 in mouse testis is considerably higher than that in other tissues. ALKBH5 silencing in mice results in testicular atrophy, lighter weight, reduced sperm quantity and quality and also causes reproductive dysfunction, such as apoptosis and fertility decline. Drosophila melanogaster lacking m^6^A modification induces fly failure and fertility decline, accompanied by the deficiencies in nerve, sex determination and dose compensation 73, 76. ALKBH5 amplification in testis is indispensable for the correct splicing of long 3'UTR of mRNA in spermatocytes, which is involved in spermatogenesis and mouse reproduction 7, 77. Inhibiting ALKBH5 results in aberrant splicing and shorter transcripts of mRNAs, ultimately developing meiosis abnormity of spermatocytes and male infertility in mice 77, 78. Meclofenamic acid suppresses cell viability and blocks G1/S progression in spermatogonia of mice through accelerating the degradation of CDK2 mRNA via specifically decreasing FTO and elevating the m^6^A content of 3'UTR 79.

YTHDF2-mediated mRNA degradation exerts essential functions in sexual reproduction of mammalian 80, 81. YTHDF2 deletion in an embryo of zebrafish decelerates the degradation of m^6^A- modified maternal transcripts and impedes the activation of zygote genome, which prevents the maternal-to-zygotic transition, thereby delaying the development of zebrafish larvae 82. YTHDF2 acts as an intrinsic determinant for early zygotic development and mammalian egg quality 81. Due to the failure of regulating the transcription of genes involved in oocyte maturation, female mice with YTHDF2 knockout are deprived of fertility 81. YTHDC1 is essential for oocyte growth and maturation in female mice and spermatogonia survival in male mice, and m^6^A methylation can compromise the alternative splicing defects caused by YTHDC1 silencing in mouse oocytes 37. In the testis of wild male mice, YTHDC2 expression is up-regulated at the beginning of meiosis, while in the testis with YTHDC2 knockout, healthy sperm could not be produced 82.

### m^6^A in virus replication and infection

m^6^A methylation not only works a crucial role in host cells; but also in diverse viral RNAs. It was initially described in IAV and adenovirus 83, 84. Subsequently, it was discovered in HSV-1, KSHV, Rous sarcoma virus, avian sarcoma virus B77, feline leukemia virus, HIV-1, Zika virus (ZIKV), simian virus 40 (SV40), HBV, HCV, flavivirus and plant virus (alfalfa mosaic and cucumber mosaic virus) 85-89. m^6^A modification may present an entirely opposite pattern in the replication of different viruses. Mechanism dissection revealed that m^6^A methylation regulates virus replication and infection, which are mainly achieved by affecting the stability of virus mRNAs or genomic RNAs. And m^6^A modification in host-viral interactions is indispensable for modulating virus replication and infection 87, 90, 91.

The translation of HIV-1 mRNA is dominated by cap-dependent and independent manners 92, and the m^6^A modification in stem-loop II region of HIV-1 Rev response element (RRE) contributes to HIV-1 Rev protein binding to RRE, thereby affecting nuclear export of HIV-1 mRNA 86. Accumulating studies have identified the specific sites of m^6^A and methylation peaks in HIV-1 mRNAs 86, 93-95, all of which suggests that m^6^A modification performs a significant role in the HIV-1 genome. Altering m^6^A regulators, METTL3, METTL14, FTO and ALKBH5, affect HIV-1 related protein expressions, such as GP120, p24, p55 Gag 93. p24 and Nef are down-regulated expression in CD4+ T cells infected with HIV-1 after CRISPR-Cas-mediated deletion of YTHDF2 87. HIV-1 infection is also amplified following the knockout of reader protein DF1-3 93. On the contrary, the enforced expression of these m^6^A readers inhibits HIV-1 replication and decrease Gag protein level. Drugs targeting m^6^A modification can be used as the development direction of antiviral drugs, such as 3-deazaadenosine (3-DAA), which has been shown to block the hydrolysis of S-Adenosylhomocysteine to degrade SAM, thus inhibiting the addition of m^6^A group to the mRNA substrate. Kennedy et al. treated HIV-1-infected CEM-SS cells with 3-DAA and found that the replication of HIV-1 is deprived, which proves the inhibitory effect of 3-DAA on HIV-1 replication 95. The 3-DAA, too, has an excellent inhibitory effect on the replication of RSV, IAV and other viruses 96, 97.

m^6^A writers and erasers are found to shuttle to the cytoplasm to modulate the m6A methylation of flavivirus RNAs 98, 99, and m^6^A modification regulates the virus-like particle production and the infections of HCV and ZIKV 98, 99. m^6^A methylation enriched in the KSHV genome might regulate alternative splicing of the ORF50 pre-mRNA and control governing switch of KSHV replication 85, 100. And knockdown of METTL3 abrogates the expression of KSHV lytic genes and halts virus production 85. m^6^A methylation accelerates SV40 replication by potentiating the translation and export of viral transcripts 101. HBV related HBs and Hbc proteins increases followed by METTL3 or METTL14 silencing; on the contrary, inhibiting FTO or ALKBH5 enforces their expression 87, 102. m^6^A methylation participates in the regulation of HBV life cycle via affecting transcript stability 87, 102. The lower m^6^A modification makes some viruses successfully evade the immune surveillance, so whether there is some mechanism that can detect these abnormal methylations and then feedback to the immune system for treatment, which needs further study.

### m^6^A in stress response

Following the heat shock, METTL3 locates in chromatin of the heat shock gene and catalyze m^6^A methylation. Nuclear YTHDF2 competes with FTO to prevent the reversal of m^6^A modification, thereby enhancing mRNA translation in a cap-dependent manner and improving the formation of related proteins 103. YTHDF1 is responsible for confining stress particles induced by protein-encapsulated mRNAs and protein particles to the nucleus, somehow abrogating protein release and mRNA expression 103. When the stress source disappears, the stress particles are re-depolymerized and released, and then encapsulated mRNAs re-express 103, which contributes to avoid the inflammation occurrence induced by stress sources whenever possible and is feasible and beneficial for injury repair. Similarly, due to be subjected to UV damage to DNA, METTL3-catalyzed m^6^A is quickly located at the injury site, and DNA polymerase κ is also recruited to ameliorate injury repair 104. Recent research has demonstrated that multivalent m^6^A motifs enhances phase separation of YTHDF1/2/3 proteins through juxtaposing low-complexity domain of YTHDFs, which causes the presence of phase-separated compartments, such as P-body, neuronal RNA and stress granules 105, 106.

### m^6^A in immune response

By regulating the IL-7/STAT5/SOCS pathway, m^6^A methylation affects T cell state, while the absence of METTL3 can destroy the homeostasis and differentiation of T cells 107. Blockade of METTL3 in immature T cells augments the expression of SOCS1, SOCS3 and CISH, which inhibit the activation STAT5 mediated by IL-7 and the proliferation and differentiation of T cells 107. Due to the loss of differentiation ability in METTL3-deficient T cells, it is insufficient to induce autoimmune diseases 107. After virus infection, DDX46 recruits ALKBH5 to erase m^6^A modification of antiviral transcripts, resulting in the inability of antiviral transcripts translating into IFN-I and the failure of initiating natural antiviral immune responses 47. m^6^A modification also participates in tumor antigen- specific immune response through modulating the translation efficiency of lysosome cathepsin in dendritic cells 108. Knockout of YTHDF1 in mice can enhance the immune response of CD8+ T cells 108. These findings are expected to provide innovative ideas for the treatment of autoimmune diseases and understanding the molecular mechanism of innate immunity in various diseases, especially virus infection 109.

### m^6^A in differentiation of stem cells

m^6^A methylation is involved in regulating the fate-determining process of stem cells 75, 110. m^6^A modification maintains the balance of gene expression between endothelial cells and hematopoietic cells during endothelial-hematopoietic transformation by regulating Notch1a mRNA stability via YTHDF2, which then affects the fate of hematopoietic stem cells 111. Reversing m^6^A modification treated with METTL3 or METTL14 blockade contributes to the amplification of transcription factors maintaining cell pluripotency and inhibits the self-renewal of embryonic stem cells 112, 113. METTL3 knockout interferes with differentiation and self-renewal of mouse embryonic stem cells via decreasing m^6^A methylation of NANOG, leading to embryonic death 75. However, ZC3H13 depletion impairs self-renewal of mouse embryonic stem cells due to the cells cultured in metastable naive condition 114. METTL3 depletion potentiates both calcium deposition and alkaline phosphatase activity of mesenchymal stem cells by modulating the expression of MYD required for activating NF-κB, as a repressor of osteogenesis, which uncovers the inhibitory role of METTL3 in osteogenic differentiation 115. ALKBH5 deteriorates the m^6^A modification of NANOG mRNA to promote its expression in breast cancer stem cells following exposure to hypoxia, which furtherly accelerates cell proliferation and regulates cell differentiation 116. It has further been proved that FTO stimulates the transformation of normal hematopoietic stem cells to pathological cells in leukemia 117, and can influence the selective splicing of key genes related adipogenesis, thereby affecting the preadipocyte differentiation 41. In conclusion, m^6^A methylation plays a key role in regulating the differentiation and development of stem cells.

### m^6^A in nervous system

Brain is one of the organs with the most abundant of m^6^A modification 118, 119. m^6^A regulatory proteins are implicated in the cerebral cortex and synaptic function, axonal regeneration, self-renewal of neural stem cells and cerebellar development 118-122. m^6^A methylation effectuates the region-specific gene regulation in the mouse brain 118. Knockdown of METTL14 in the central nervous system of mice affect the development of cerebral cortex 120, METTL3 silencing seriously affects the development of cerebral cortex and cerebellum, leading to obvious motor dysfunction, even death during lactation 123. Mechanically, m^6^A modification modulates the stability of transcripts associated with cerebellar development and affects pre-mRNA splicing in synapse 123.

Subsequent studies showed that m^6^A enrichment in the prefrontal lobe of mice contributes to learning training, implying the underlying mechanisms of m^6^A methylation in spatial learning and memory 121. Suckling mouse with METTL3 knockout in the cortex and hippocampus displays a significant decline in learning ability 124. In YTHDF1 knockout mice, hippocampus-dependent learning and memory dysfunction occurs, and the function and plasticity of excitatory synapses in hippocampal neurons are also impaired; on the contrary, forced expression of YTHDF1 ameliorates the injury 125. The crucial role of m^6^A modification lays a foundation for further exploring the pathophysiology of memory formation.

### m^6^A in cancer

Recently, emerging evidence has revealed that there are disorders of m^6^A components and abnormal modification of m^6^A in many types of cancers (Figure 4), which leads to the inactivation or overexpression of downstream carcinogenic or tumor suppressor genes and plays an important role in oncogenic transformation and tumor progression. It is worth noting that the same components in different types of tumors, or different components in the same tumor do not play the same role, or even the opposite.

m^6^A modification of mRNAs is significantly up-regulated during epithelial-mesenchymal transition, and it can regulate the genes related to invasion, metastasis and cell adhesion 126. Through database analysis, it is found that FTO can regulate EGFR-related pathways and angiogenic signals, and overexpression of FTO inhibits tumor growth *in vivo* 127. In tumor cells, METTL14 and ALKBH5 may play a cancer-promoting role by regulating the expression of angiogenesis-related genes such as TGF-β 128. METTL3 or METTL14 depletion could induce the expression of ADAM19, EPHA3 and KLF4 mRNAs and promote the growth, self-renewal and tumorigenesis of glioblastoma stem cells 128. Hypoxia stimulation-induced ALKBH5 abrogates the m^6^A methylation of NANOG mRNAs and enhances the mRNA stability, which in turn promotes the maintenance and metastasis of breast tumor stem cells 116. In breast cancer, targeted inhibition of FTO can reduce the production of lactic acid and ATP and inhibit the activities of pyruvate kinase and hexokinase, and lead to the inactivation of AKT, overexpression of FTO can promote the glycolysis ability of tumor cells and the activation of PI3K/AKT pathway 129. m^6^A modification activates Wnt/β-catenin pathway by stabilizing FZD10, which promotes BRAC deficiency and induce PARP inhibitor resistance of epithelial ovarian cancer cells 130. Blockade of METTL3 can significantly increase the sensitivity of pancreatic cancer cells to anticancer drugs 5-fluorouracil, cisplatin and radiotherapy 51. In addition, R-2-hydroxyglutarate (R-2HG) can obviate FTO activity, which augments the m^6^A modification in R-2HG-sensitive leukemic cells, and then reduce the stability of MYC/CEBPA transcripts, promoting cell cycle arrest and apoptosis 131. m^6^A modification is implicated in various aspects of multiple malignancies via regulating differentiation, proliferation, apoptosis, invasion, migration, angiogenesis, energy metabolism, autophagy and chemoradiotherapy resistance. Uncovering the underlying mechanism of m^6^A modification in initiation and progression of cancer extends opportunities for molecular pathological diagnosis and therapy strategies in cancer.

### m^6^A in heart

Enhanced METTL3 induce cardiomyocyte hypertrophy without additional stimulation, and inhibition of METTL3 can effectively block the occurrence of hypertrophy *in vitro* 132, 133. m^6^A methylation can expedite adaptive growth of cardiomyocytes and induce spontaneous cardiomyocyte remodeling 132, 133. Studies have found that FTO is associated with various heart defects, including hypertrophic cardiomyopathy and interventricular defects, septal and atrioventricular defects, arrhythmias and coronary heart disease 134. Knockdown of FTO can cause the disorder of calcium homeostasis and sarcomere dynamics in heart failure and hypoxic cardiomyocytes and decrease the contractile function of cardiomyocytes 135.

## Conclusions

The milestone in the study of RNA epigenetics was discovered in 2011 by He et al, who discovered the first m^6^A demethylase FTO, which reveals that m^6^A modification is dynamic and reversible, and enriches the post-transcriptional modification function of RNA and generates refueled passion in m^6^A research. In this review, m^6^A enzymes and its regulation on RNA processing and metabolism, various biological processes as well as in pathogenesis have been revealed. m^6^A modification can functionally regulate the transcriptome of eukaryotes, such as mRNA splicing, nucleation, localization, translation and stabilization and play crucial roles in various biological processes, such as stem cell differentiation, T cell homeostasis, brain development, biological rhythm, spermatogenesis, as well as the occurrence of a variety of diseases, including tumor, obesity and infertility. Therefore, it is particularly important to study the dynamic modification of m^6^A and the specific molecular mechanism of how it affects the biological function of cells.

However, the other components of m^6^A methyltransferase complex are not clear, the tissue-specific demethylase remains to be explored, and the binding proteins mediating distinct functions also need to be further discovered. The single-base, high-resolution and high-throughput sequencing technology for detecting m^6^A methylation also needs to be improved. Regulatory mechanisms and detailed biological functions of m^6^A modification need to be further investigated.

## Abbreviations

ALKBH5: AlkB homolog 5
METTL3: methyltransferase-like protein 3
m^6^A: N6-methyladenosine
FTO: fat-mass and obesity-associated protein
RRE: Rev response element
R-2HG: R-2-hydroxyglutarate
WTAP: Wilms Tumor 1 Associated Protein
YTH: YT521-B homology
3-DAA: 3-deazaadenosine
3' UTRs: 3' untranslated regions
